# Report of Deaths in Buffalo for the Month of January, 1855

**Published:** 1855-03

**Authors:** J. Root

**Affiliations:** Health Physician


### ART. VII.—*Report of Deaths in Buffalo for the month*

| DISEASES. | Males. | Females. | Total. | AGE. 1 year and under. Male. | Female. | 1 to 2 years. Male. | Female. |
|---|---|---|---|---|---|---|---|
| Apoplexia, | 2 | .... | 2 | 1 | .. | 1 | .. |
| Atrophia, | 1 | .... | 1 | 1 | .. | .. | .. |
| Croup, | 3 | 3 | 6 | .. | 2 | 3 | .. |
| Consumption, | 13 | 8 | 21 | .. | .. | 2 | .. |
| Child Bed, | .... | 1 | 1 | .. | .. | .. | .. |
| Convulsions, | 5 | 3 | 8 | 2 | 2 | 2 | .. |
| Chronic Bronchitis, | 1 | 1 | 2 | .. | .. | .. | .. |
| Congestion of Lungs, | .... | 2 | 2 | .. | 2 | .. | .. |
| " " Brain, | 1 | .... | 1 | .. | .. | .. | .. |
| Cancer, | .... | 1 | 1 | .. | .. | .. | .. |
| Diarrhœa, | 2 | 1 | 3 | 1 | .. | 1 | 1 |
| Dentition, | .... | 2 | 2 | .. | 1 | .. | 1 |
| Dropsy, | 1 | .... | 1 | .. | 1 | .. | .. |
| Debility, | 4 | 1 | 5 | 2 | 1 | .. | .. |
| Dysentery, | .... | 2 | 2 | .. | 1 | .. | .. |
| Disease of Heart, | .... | 1 | 1 | .. | .. | .. | 1 |
| Fever, | 1 | 1 | 2 | .. | .. | .. | .. |
| " Typhoid, | 4 | .... | 4 | .. | .. | .. | .. |
| " Typhus, | 5 | 1 | 6 | .. | .. | .. | .. |
| " Scarlet, | 3 | 4 | 7 | .. | .. | 1 | 1 |
| (" Nervous,) | .... | 1 | 1 | .. | .. | .. | .. |
| Gangrene of Lungs, | 1 | .... | 1 | .. | .. | .. | .. |
| Hydrocephalus, | 3 | 2 | 5 | 2 | .. | 1 | .. |
| Inflammation of Brain, | 3 | .... | 3 | 2 | .. | .. | .. |
| " " Bowels, | .... | 2 | 2 | .. | .. | .. | .. |
| " " Bladder, | 1 | .... | 1 | .. | .. | .. | .. |
| Masturbation, | 1 | .... | 1 | .. | .. | .. | .. |
| Old Age, | 3 | 6 | 9 | .. | .. | .. | .. |
| Pleurisy, | .... | 1 | 1 | .. | .. | .. | .. |
| Pneumonia, | 4 | 6 | 10 | 1 | 1 | 1 | 2 |
| Pulmonary Apoplexia, | 2 | .... | 2 | .. | .. | 1 | .. |
| Quinsey, | 1 | .... | 1 | 1 | .. | .. | .. |
| Still-born, | 4 | 3 | 7 | .. | .. | .. | .. |
| Syncope, | 5 | 3 | 8 | 3 | 2 | .. | 1 |
| Unknown, | .... | 1 | 1 | .. | .. | .. | .. |
| Whooping Cough, | .... | 2 | 2 | .. | 1 | .. | .. |
| Totals, | 74 | 58 | 132 | 9 | 4 | 4 | 4 |

NATIVITIES.—American, 46. German, 43. Irish, 19. English, 5.

*of January*, 1855. By J. Root, M. D., Health Physician.

| AGE. | | | | | | | | | | | | | | | | | | | | | | | | | | | | | | | |
|---|---|---|---|---|---|---|---|---|---|---|---|---|---|---|---|---|---|---|---|---|---|---|---|---|---|---|---|---|---|---|---|
| 2—5 | | 5—10 | | 10—15 | | 15—20 | | 20—25 | | 25—30 | | 30—40 | | 40—50 | | 50—60 | | 60—70 | | 70—80 | | 80—90 | | 90—100 | | 100 and upward. | | Not given. | |
| Male. | Female. | Male. | Female. | Male. | Female. | Male. | Female. | Male. | Female. | Male. | Female. | Male. | Female. | Male. | Female. | Male. | Female. | Male. | Female. | Male. | Female. | Male. | Female. | Male. | Female. | Male. | Female. | Male. | Female. |
| -- | -- | -- | -- | -- | -- | -- | -- | -- | -- | -- | -- | -- | -- | -- | -- | -- | -- | -- | -- | -- | -- | -- | -- | | | | | | |
| -- | -- | -- | -- | -- | -- | -- | -- | -- | -- | -- | -- | -- | -- | -- | -- | -- | -- | -- | -- | -- | -- | -- | -- | | | | | | |
| 1 | -- | -- | -- | -- | -- | -- | -- | -- | -- | -- | -- | -- | -- | -- | -- | -- | -- | -- | -- | -- | -- | -- | -- | | | | | | |
| -- | -- | -- | 1 | -- | -- | -- | -- | 1 | -- | 5 | -- | 3 | 4 | 1 | 1 | -- | 2 | -- | 1 | -- | -- | -- | -- | | | | | | |
| -- | -- | -- | -- | -- | -- | -- | -- | -- | -- | -- | -- | -- | 1 | -- | -- | -- | -- | -- | -- | -- | -- | -- | -- | | | | | | |
| -- | -- | -- | -- | -- | -- | -- | -- | -- | -- | -- | 1 | -- | -- | -- | -- | -- | -- | -- | -- | -- | -- | -- | -- | | | | | | |
| -- | -- | -- | -- | -- | -- | -- | -- | -- | -- | -- | -- | -- | -- | -- | -- | -- | -- | 1 | 1 | -- | -- | -- | -- | | | | | | |
| -- | -- | -- | -- | -- | -- | -- | -- | -- | -- | -- | -- | -- | -- | -- | -- | -- | -- | -- | -- | -- | -- | -- | -- | | | | | | |
| -- | -- | -- | -- | -- | -- | -- | -- | -- | -- | -- | -- | 1 | -- | -- | -- | -- | -- | -- | -- | -- | -- | -- | -- | | | | | | |
| -- | -- | -- | -- | -- | -- | -- | -- | -- | -- | -- | -- | -- | -- | -- | -- | -- | 1 | -- | -- | -- | -- | -- | -- | | | | | | |
| -- | -- | -- | -- | -- | -- | -- | -- | -- | -- | -- | -- | -- | -- | -- | -- | -- | -- | -- | -- | -- | -- | -- | -- | | | | | | |
| -- | -- | -- | -- | -- | -- | -- | -- | -- | -- | -- | -- | -- | -- | -- | -- | -- | -- | -- | -- | -- | -- | -- | -- | | | | | | |
| -- | -- | -- | -- | -- | -- | -- | -- | -- | -- | -- | -- | -- | -- | -- | -- | -- | -- | -- | -- | -- | -- | -- | -- | | | | | | |
| 1 | -- | -- | -- | -- | -- | -- | -- | -- | -- | -- | -- | -- | -- | -- | -- | 1 | -- | -- | -- | -- | -- | -- | -- | | | | | | |
| -- | -- | -- | -- | -- | -- | -- | -- | -- | -- | -- | -- | -- | 1 | -- | -- | -- | -- | -- | -- | -- | -- | -- | -- | | | | | | |
| -- | -- | -- | -- | -- | -- | -- | -- | -- | -- | -- | -- | -- | -- | -- | -- | -- | -- | -- | -- | -- | -- | -- | -- | | | | | | |
| 1 | -- | -- | -- | -- | -- | -- | -- | -- | -- | -- | -- | -- | 1 | -- | -- | -- | -- | -- | -- | -- | -- | -- | -- | | | | | | |
| -- | -- | -- | -- | -- | -- | -- | -- | -- | -- | 2 | -- | 1 | -- | -- | -- | 1 | -- | -- | -- | -- | -- | -- | -- | | | | | | |
| -- | -- | -- | -- | -- | -- | -- | -- | 3 | -- | -- | 1 | 1 | -- | 1 | -- | -- | -- | -- | -- | -- | -- | -- | -- | | | | | | |
| 1 | 1 | 1 | 1 | -- | 1 | -- | -- | -- | -- | -- | -- | -- | -- | -- | -- | -- | -- | -- | -- | -- | -- | -- | -- | | | | | | |
| -- | -- | 1 | -- | -- | -- | -- | -- | -- | -- | -- | -- | -- | -- | -- | -- | -- | -- | -- | -- | -- | -- | -- | -- | | | | | | |
| -- | -- | -- | -- | -- | -- | -- | -- | -- | -- | -- | -- | 1 | -- | -- | -- | -- | -- | -- | -- | -- | -- | -- | -- | | | | | | |
| -- | 2 | -- | -- | -- | -- | -- | -- | -- | -- | -- | -- | -- | -- | -- | -- | -- | -- | -- | -- | -- | -- | -- | -- | | | | | | |
| 1 | -- | -- | -- | -- | -- | -- | -- | -- | -- | -- | -- | -- | -- | -- | -- | -- | -- | -- | -- | -- | -- | -- | -- | | | | | | |
| -- | 1 | -- | -- | -- | -- | -- | -- | -- | -- | -- | -- | -- | 1 | 1 | -- | -- | -- | -- | -- | -- | -- | -- | -- | | | | | | |
| -- | -- | -- | -- | -- | -- | -- | -- | -- | -- | -- | -- | -- | -- | -- | -- | -- | -- | -- | -- | 1 | -- | -- | -- | | | | | | |
| -- | -- | -- | -- | -- | -- | -- | -- | -- | -- | -- | -- | -- | -- | -- | -- | -- | -- | -- | -- | -- | -- | -- | -- | | | | | | |
| -- | -- | -- | -- | -- | -- | -- | -- | -- | -- | -- | -- | -- | -- | -- | -- | 1 | 1 | -- | 1 | 2 | 3 | -- | 1 | | | | | | |
| -- | -- | -- | -- | -- | -- | -- | -- | -- | 1 | -- | -- | -- | -- | -- | -- | -- | -- | -- | -- | -- | -- | -- | -- | | | | | | |
| -- | -- | -- | -- | -- | -- | -- | 1 | -- | 2 | -- | -- | 1 | -- | 1 | -- | -- | -- | -- | -- | -- | -- | -- | -- | | | | | | |
| -- | -- | -- | -- | -- | -- | -- | -- | -- | -- | 1 | -- | -- | -- | -- | -- | -- | -- | -- | -- | -- | -- | -- | -- | | | | | | |
| -- | -- | -- | -- | -- | -- | -- | -- | -- | -- | -- | -- | -- | -- | -- | -- | -- | -- | -- | -- | -- | -- | -- | -- | | | | | | |
| -- | -- | -- | -- | -- | -- | -- | -- | -- | -- | -- | -- | -- | -- | -- | -- | -- | -- | -- | -- | -- | -- | -- | -- | | | | | | |
| -- | -- | 1 | -- | -- | -- | -- | -- | -- | -- | -- | -- | -- | -- | 1 | -- | -- | -- | -- | -- | -- | -- | -- | -- | | | | | | |
| -- | -- | -- | -- | -- | -- | -- | -- | -- | -- | -- | -- | -- | 1 | -- | -- | -- | -- | -- | -- | -- | -- | -- | -- | | | | | | |
| -- | 1 | -- | -- | -- | -- | -- | -- | -- | -- | -- | -- | -- | -- | -- | -- | -- | -- | -- | -- | -- | -- | -- | -- | | | | | | |
| 2 | 5 | 2 | 2 | — | 1 | — | 1 | 3 | 3 | 3 | 1 | 4 | 2 | 3 | — | 2 | 1 | — | 1 | 3 | 3 | — | 1 | — | — | — | — | — | — |

French, 4. Scotch, 1. Colored, 2. Unknown, 12.

